# Alkylation of 2,4-(1*H*,3*H*)-Quinazolinediones with Dialkyl Carbonates Under Microwave Irradiations

**DOI:** 10.3390/molecules14051860

**Published:** 2009-05-20

**Authors:** Ignacio Alfredo Rivero, Leticia Guerrero, Karla Alejandra Espinoza, Martha Cecilia Meza, Jesús Ramón Rodríguez

**Affiliations:** 1 Centro de Graduados e Investigación en Química, Instituto Tecnológico de Tijuana. C.P. 1166. Tijuana, B.C. 22000, Mexico; 2 Instituto Nacional de Investigaciones Nucleares, Departamento de Química. Carretera México Toluca S/N, La Marquesa, Ocoyoacac, Mexico, D.F. C.P. 52750; 3 Facultad de Química, Universidad Autónoma de Baja California, Calzada Tecnológico #14418, Mesa de Otay. Tijuana, B.C, Mexico, C.P. 22390

**Keywords:** alkylation, quinazoline-2,4-dione, antihypertensive activity, microwaves

## Abstract

Alkylation is a very important chemical reaction which modifies the biological properties of drugs. Quinazolinedione derivatives are of considerable interest due to their wide array of pharmacological properties. We now report application of a practical alkylation procedure to several quinazolinediones, including pelanserine (**5f**), which shows antihypertensive properties, 1‑methyl-3-(2'-phenylethyl)-1*H*,3*H*-quinazoline-2,4-dione (**1ab**) and 1-methyl-3-[2'-(4'-methoxyphenyl)ethyl]-l*H*,3*H*-quinazoline-2,4-dione (**1ae**), which had been isolated from natural sources. The alkylation was optimized using dimethyl and diethyl carbonates under microwave irradiations.

## Introduction

In general alkylation involves substitution of a hydrogen atom by an alkyl group and is a popular and fundamental process in organic synthesis. Several functional groups as α-carbon [1], alcohols [2], amines [3], carboxylic acids [4] and amides-NH [5] are protected by alkylation reactions. These modifications change the physic chemical and biological properties of such compounds. Our group has been working on the synthesis of quinazolinone and quinazolinedione derivatives, which are of considerable interest because of their wide array of pharmacological properties [6,7,8,9,10,11,12,13,14,15,16,17,18,19,20]. We have synthesized heterocycles containing the quinazoline-2,4-dione backbone, which are known to exhibit potential anti-hypertensive properties [21,22,23,24,25]. We have described the synthesis of pelanserine (**5f**), a potent anti-hypertensive agent [26], and a several quinazoline-2,4-diones with amino acids and dipeptide, which when tested showed mild to no antihypertensive properties [27]. 

Recently, we synthesized two alkaloids containing the quinazoline-2,4-dione ring skeleton – 1‑methyl-3-(2'-phenylethyl)-1*H*,3*H*-quinazoline-2,4-dione (**1ab**) and 1-methyl-3-[2'-(4'-methoxy- phenyl)ethyl]-l*H*,3*H*-quinazoline-2,4-dione (**1ae**) – which have been isolated from the seed husks of *Zanthoxylum arborescens* [28]. Alkylation reactions were performed with methyl iodide and TMG as a base, but the reagents are expensive *abd* toxic and take about one hour at 55^0^C to complete the reaction [29]. Herein, we propose a novel methodology for the alkylation using inexpensive dimethyl and diethyl carbonates which are very stable liquids, non-reactive under normal conditions. The reactions were assisted and optimized by microwaves, taking only a few minutes to complete. This efficient process was applied to a quinazolinedione library to improve the methodology without toxic reagents.

**Figure 1:**
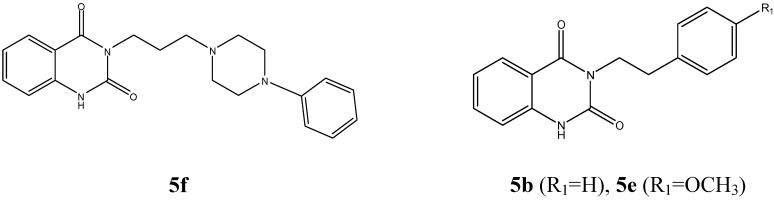
Examples of quinazoline-2,4- diones: Pelanserine (**5f**) a potent anti-hypertensive agent, and two natural products [**5**(**b,e**)].

## Results and Discussion

**Scheme 1:**
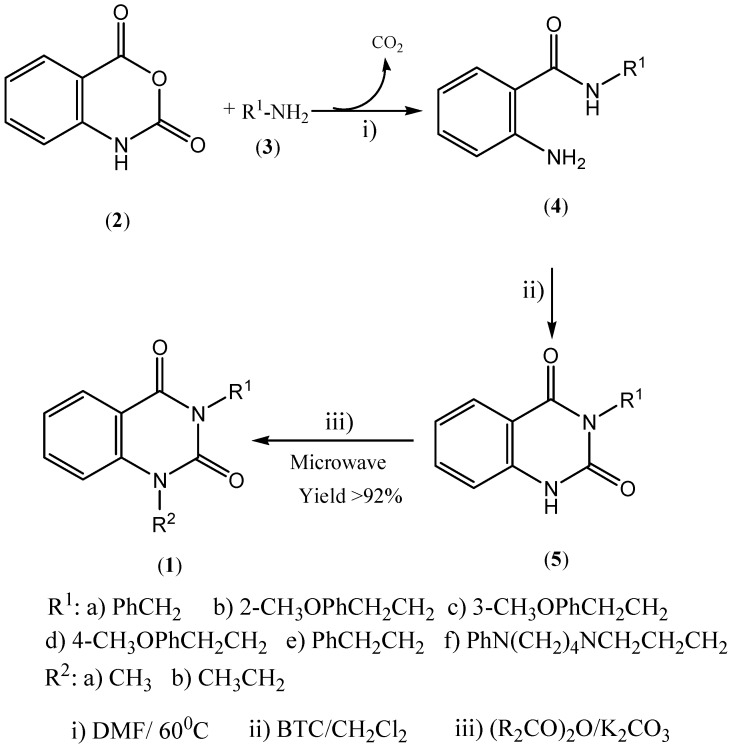
Steps to obtain to the N-methyl quinazoline-2,4-diones.

Alkylation of *NH*-containing heteroaromatic compounds is an important transformation that regularly employs toxic and hazardous reagents such as methyl iodide [29] or dimethyl sulfate [30]. Dialkyl carbonates are an attractive alternative as alkylating reagents for *NH*-containing heteroaromatic compounds. Quinazoline-2,4-diones **5**(**a,b,c,d,e,f**) were thus prepared using our methodology [26]. Initially, we prepared the *ortho*-aminobenzamides from the reactions of isatoic anhydride with amines and the cyclization was carried out with bis(trichloride methyl)carbonate (BTC, triphosgene). Finally, the alkylation with dimethyl carbonate was optimized under microwave irradiation and the conditions were fixed at 200 W, 130 °C, for 15 minutes, using K_2_CO_3_ as base (Scheme 1). The reaction was filtered, to get a >94% yield. The alkylation with diethyl carbonate was similar, in this case it was necessary to increase the temperature to 160 °C to obtain the ethyl-quinazoline-2,4-diones in a >92% yield. Ethylation products had to be purified by column chromatography on silica gel to remove the excess of diethyl carbonate. By applying microwave irradiation further rate enhancements were accomplished. 

The new methodology has the advantages of rapid reaction times, ease of operation and purification and the use of readily available reagents, and the avoidance of toxic alkylating reagents. In this work methyl and ethyl quinazoline-2,4-dione libraries were prepared, which are detailed in Table 1. The yields obtained with the optimized method were excellent, with conversions of 92-98%. Therefore, this is a very efficient method, with easy purification of products, since no by-products were observed.


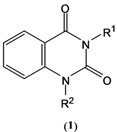


**Table 1:** Alkylated quinazoline-2,4-diones.

Entries	R^1^	R^2^	% Yield
**1aa**	PhCH_2_	CH_3_	94
**1ab**	PhCH_2_CH_2_	CH_3_	98
**1ac**	2-CH_3_OPhCH_2_CH_2_	CH_3_	95
**1ad**	3-CH_3_OPhCH_2_CH_2_	CH_3_	96
**1ae**	4-CH_3_OPhCH_2_CH_2_	CH_3_	98
**1af**	PhN(CH_2_)_4_NCH_2_CH_2_CH_2_	CH_3_	93
**1ba**	PhCH_2_	CH_3_CH_2_	96
**1bb**	PhCH_2_CH_2_	CH_3_CH_2_	96
**1bc**	2-CH_3_OPhCH_2_CH_2_	CH_3_CH_2_	95
**1bd**	3-CH_3_OPhCH_2_CH_2_	CH_3_CH_2_	94
**1be**	4-CH_3_OPhCH_2_CH_2_	CH_3_CH_2_	97
**1bf**	PhN(CH_2_)_4_NCH_2_CH_2_CH_2_	CH_3_CH_2_	92

## Conclusions

We have developed a simple method to methyl or ethyl alkylation of amide-*NH* functions with dialkyl carbonates, which were assisted by microwave irradiation. We used several quinazoline-2,4-diones which were previously synthesized by our group for biological evaluation as potential antihypertensive agents. We have proven that this method is very fast, clean, with almost complete conversion, using stable reagents avoiding possible contamination. We are currently exploring this reaction without solvent, as a green chemistry process. The amounts of K_2_CO_3_ are important which were established at three equivalents, thus working in a more efficient way. After several experiments, optimum conditions were determined. The basic backbone provides a source for introduction of different heterocyclic extension on the amide –*NH,* in order to diversify the quinazoline-2,4-dione structural system.

## Experimental

### General

Melting points were measured on an Electrothermal 88629 apparatus and are uncorrected. Infrared (IR) spectra were recorded on a Perkin Elmer FT-IR 1600 spectrometer. ^1^H-NMR and ^13^C-NMR spectra were recorded at 200 MHz and 50.289 MHz, respectively, on a Varian Mercury 200 spectrometer in CDCl_3_ with TMS as internal standard. Mass spectra were obtained on an Agilent 1100 series LC/MSD Trap, SL Spectrometer by electrospray insertion. Microwave equipment was a self-tuning single mode CEM Discover^TM^ Focused Synthesizer.

### General Method for Methylation of Quinazoline-2,4-diones ***4***

*1-Methyl-3-(2'-phenylethyl)-1H,3H-quinazoline-2,4-dione* (**1ab**)*.* Dimethyl carbonate (1.25 mL) was added to a solution of 3-phenylethyl-1H-quinazoline-2,4-dione (**5b, **0.125g, 0.38 mmol) in DMF (1.25 mL) and K_2_CO_3_ (3 equiv) as base. The mixture was placed in a microwave reactor vessel (10 mL) and heated at 130 °C for 15 minutes and cooled to RT, then diluted with CH_2_Cl_2_ and H_2_O. The aqueous layer was removed, and the organic layer was washed with H_2_O, twice with 2 M HCl or 10% aqueous citric acid, twice with saturated aqueous NaHCO_3_, and twice with H_2_O. The organic layer was dried over anhydrous Na_2_SO_4_, filtered, and concentrated under vacuum to afford **1ab** as a white solid. Yield >98 %; mp 99-101°C (Lit. [25], mp. 100-102°C); IR (KBr): 3042, 2929, 1701, 1654, 1610, 1481 cm^-1^; ^1^H-NMR: δ 8.23 (dd, 1H, *J_1_* =1.7, *J_2_* =7,8 Hz, Ar-H), 7.65 (ddd, 1H, *J_1_* = 1,7, *J_2_* =7,3, *J_3_* =8,4 Hz, Ar-H), 7.34-7.16 (m, 7H, Ar-H), 4.29 (ddd, *J_1_* = 5.6, *J_2_* = *J_3_* =7.8 Hz, 2H, N-CH_2_), 3.57 (s, 3H, N-CH_3_), 2.96 (ddd, J_1_ = 5.2, J_2_ = J_3_ =8,0 Hz, Ar-CH_2_) ppm; ^13^C-NMR: δ 161.3, 150.5, 140.2, 138.3, 134.8, 129.9, 128.7, 128.6, 128.2, 126.2, 122.7, 120.9, 113.3, 43.3, 33.9, 30.6 ppm; ESI-MS (m/e): 280.1[M+ H]^+^.

### The following compounds were prepared in similar fashion:

*1-Methyl-3-[2'-(2'-methoxyphenyl)ethyl]-lH,3H-quinazoline-2,4-dione* (**1ac**). White solid. Yield >95%; mp 155-163°C; IR (KBr): 2943, 1703, 1651, 1608, 1484, 1243, 1028 cm^-1^;^ 1^H-NMR: δ 8.21 (dd, 1H, *J_1_* =1.6, *J_2_* =7.9 Hz, Ar-H), 7.66 (ddd, 1H, *J_1_* = 1.7, *J_2_* =7.3, *J_3_* =8.5 Hz, 1H, Ar-H), 7.28-7.16 (m, 5H, Ar-H), 6.85(ddd, 1H, *J_1_* = 1.0, *J_2_* = *J_3_* =8.2 Hz, Ar-H), 4.33 (ddd, 2H, *J_1_* = 5.6, *J_2_* = *J_3_* =7.4 Hz, Ar-CH_2_), 3.81 (s, 3H, O-CH_3_), 3.58(s, 3H, N-CH_3_), 3.02 (ddd, 2H, *J_1_* = 5.8, *J_2_* = *J_3_* =7.4 Hz, Ar-CH_2_) ppm; ^13^C-NMR: δ 161.3, 157.8, 140.2, 134.9, 130.6, 128.6, 127.7, 127.1, 122.8, 120.4, 113.4, 110.2, 55.3, 41.8, 30.6, 28.7 ppm; ESI-MS (m/e): 310.9[M+ H]^+^; 332 [M +Na]^+^.

*1-Methyl-3-[2'-(3'-methoxyphenyl)ethyl]-lH,3H-quinazoline-2,4-dione* (**1ad**). White solid. Yield 96%; mp 131-133°C; IR (KBr): 2945, 2833, 1699, 1656, 1604 cm^-1^; ^1^H-NMR: δ 8.23 (dd, 1H, *J_1_* =1.7, *J_2_* =7.9 Hz, Ar-H), 7.63 (ddd, 1H, *J_1_* =1.6, *J_2_* =7.2, *J_3_* =8.4 Hz, Ar-H), 7.25-7.15 (m, 4H, Ar-H), 6.78 (ddd, 1H, *J_1_* =1.0, *J_2_* =2.5, *J_3_* =8.2 Hz, Ar-H), 4.30 (ddd, 2H, *J_1_* = 5.4, *J_2_* = *J_3_* =7.6 Hz, N-CH_2_), 3.79 (s, 3H, O-CH_3_), 3.55 (s, 3H, N-CH_3_), 2.98 (ddd, 2H, *J_1_* =5.1, *J_2_* = *J_3_* =7.6 Hz, Ar-CH_2_) ppm; ^13^C-NMR: δ 162.5, 159.9, 148.5, 140.5, 132.8, 132.4, 129.7, 127.0, 126.4, 122.7, 117.4, 116.8, 116.3, 112.1, 55.1, 40.7, 35.7 ppm; ESI-MS (m/e): 332.9 [M +Na]^+^

*1-Methyl-3-[2'-(4'-methoxyphenyl)ethyl]-lH,3H-quinazoline-2,4-dione* (**1ae**). White solid. Yield >98%; mp. 134-136°C. (Lit. [25] mp. 133-134°C); IR (KBr): 3301, 2928, 1700, 1647, 1600, 1400, 1261 cm^-1^; ^1^H‑NMR: δ 8.21 (dd, 1H, *J_1_* =1.6, *J_2_* =7.8 Hz, Ar-H), 7.69 (ddd, 1H, *J_1_* = 1.7, *J_2_* =7.4, *J_3_* =8.5 Hz, Ar-H), 7.30-7.18 (m, 6H, Ar-H), 6.80 (ddd, 1H, *J_1_* =1.0, *J_2_* =2.5, *J_3_* =8.2 Hz, Ar-H), 4.26 (ddd, 2H, *J_1_* = 5.2, *J_2_* = *J_3_* =7.8 Hz, N-CH_2_), 3.80 (s, 3H, O-CH_3_), 3.59 (s, 3H, N-CH_3_), 2.95 (ddd, 2H, *J_1_* = 5.2, *J_2_* = *J_3_* =7.8 Hz, Ar-CH_2_) ppm; ^13^C-NMR: δ 161.8, 158. 2, 140.5, 130.1, 129.2, 124.2, 114.0, 113.6, 55.5, 43.7, 33.4, 31.0 ppm; ESI-MS (m/e): 332.9 [M +Na]^+^.

*1-Methyl-3-(benzyl)-1H,3H-quinazoline-2,4-dione* (**1aa**). White solid. Yield 94%; mp.103-106°C; IR (KBr): 3416, 2918, 1700, 1652, 1604, 1480, 1266, cm^-1^; ^1^H-NMR: δ 8.22 (dd, 1H, *J_1_* =1.6, *J_2_* =7.9 Hz, Ar-H), 7.64 (ddd, 1H, *J_1_* =1.6, *J_2_* =7.3, *J_3_* =8.5 Hz, Ar-H), 7.52 (dd, 2H, *J_1_* =1.7, *J_2_* =7.8 Hz, Ar-H), 7.34-7.13 (m, 5H, Ar-H), 5.27 (s, 2H, N-CH_2_), 3.57 (s, 3H, N-CH_3_) ppm;^ 13^C-NMR: δ 161.7, 150.9, 140.9, 137.0, 135.1, 129.7, 129.0, 128.3, 127.5, 122.9, 115.5, 113.5, 44.9, 30.7 ppm; ESI-MS (m/e) 288.9 [M+Na]^+^.

*1Methyl-3-(3-(4-phenylpiperazin-1-yl)propyl)-*1*H,*3*H-quinazoline-2,4-dione* (**1af**). White solid. Yield >93%; IR (KBr): 3018, 2932, 2880, 2803, 1695, 1647, 1604, 1223, 1110 cm^-1^. ^1^H-NMR: δ 8.18 (dd, 1H, *J_1_* =1.6, *J_2_* = 7.9 Hz, Ar-H), 7.62 (ddd, 1H, *J_1_* =1.6, *J_2_* =7.4, *J_3_* =8.4 Hz, Ar-H), 7.25-7.11 (m, 3H, Ar-H), 6.88-6.75 (m, 3H, Ar-H), 4.16 (m, 2H, N-CH_2_), 3.58 (s, 1H, N-CH_3_), 3.10 (m, 4H, N-CH_2_), 2.55 (m, 6H, N-CH_2_), 1.95 (dd, 3H, *J_1_* =7.1, *J_2_* =14.3 Hz, CH_2_) ppm; ^13^C-NMR: δ 161.8, 151.3, 148.8, 140.5, 135.0, 129.1, 128.9, 128.8, 122.8, 119.5, 115.9, 112.8, 56.1, 55.2, 53.0, 49.1, 40.5, 30.6, 24.8 ppm; ESI-MS (m/e): 392.2 [M+ H]^+^.

### General Method for Ethylation of 2,4 Quinazoline-2,4-diones ***4***

*1-Ethyl-3-(2'-phenylethyl)-1H,3H-quinazoline-2,4-dione* (**1bb**)*.* Diethyl carbonate (1.25 mL), was added to a solution of 3-phenylethyl-1-H-quinazoline-2,4-dione (**5b) (**0.125g, 0.38 mmol) in DMF (1.25 mL) and K_2_CO_3_ (3 equiv) as base. The mixture was placed in a microwave reactor vessel (10 mL) and heated at 160 °C for 15 minutes and cooled to RT, then diluted with CH_2_Cl_2_ and washed with NaCl to remove DMF. The organic layer was dried over anhydrous Na_2_SO_4_, filtered, and concentrated under vacuum. The crude was purified by silica gel column chromatography with silica using gel first hexane (60 mL) and then EtOAc (60 mL) to give **1bc** as a yellow viscous liquid. Yield >96%; IR (NaCl): 3033, 2929, 1705, 1657, 1609, 1483, 1402, 1229 cm^-1^; ^1^H-NMR: δ 8.25 (dd, 1H, *J_1_* =1.7, *J_2_* =7.8 Hz, Ar-H), 7.68 (ddd, 1H, *J_1_* = 1.7, *J_2_* =7.3, *J_3_* =8.4 Hz, Ar-H), 7.34-7.20 (m, 7H, Ar-H), 4.31 (ddd, *J_1_* = 5.6, *J_2_* = *J_3_* =7.8 Hz, 2H, N-CH_2_), 4.20 (q, 2H, *J* =7.1 Hz, -CH_2_), 2.98 (ddd, 2H, *J_1_* = 5.2, *J_2_* = *J_3_* =8.0 Hz, Ar-CH_2_), 1.34(t, 3H, *J* =7.1 Hz, -CH_3_) ppm; ^13^C-NMR: δ 161.6, 150.4, 139.5, 138.6, 135.0, 129.1, 129.0, 128.4, 126.4, 122.7, 115.8, 113.3, 43.2, 38.7, 34.0, 12.5 ppm; ESI-MS (m/e): 294.1[M+ H]^+^.

### The following compounds were prepared in similar fashion:

*1-Ethyl-3-(benzyl)-1H,3H-quinazoline-2,4-dione* (**1ba**). White solid. Yield >96%; mp. 103-105 °C; IR (NaCl): 2974, 2922, 1701, 1657, 1605, 1483 cm^-1^; ^1^H-NMR: δ 8.26 (dd, 1H, *J_1_* =1.7, *J_2_* =7.8 Hz, Ar-H), 7.66 (ddd, 1H, *J_1_* = 1.7, *J_2_* =7.3, *J_3_* =8.4 Hz, Ar-H), 7.52 (dd, 2H, *J_1_* =1.8, *J_2_* =7.9 Hz, Ar-H), 7.35-7.18 (m, 5H, Ar-H), 5.28 (s, 2H, N-CH_2_) , 4.20 (q, 2H, *J* =7.1 Hz, -CH_2_), 1.34 (t, 3H, *J* =7.1 Hz, -CH_3_) ppm; ^13^C-NMR: δ 161.4, 150.3, 139.6, 137.0, 135.1, 129.3, 129.0, 128.4, 127.5, 122.7, 120.2, 113.3, 44.9, 38.8, 12.5 ppm; ESI-MS (m/e): 280.1[M+ H]^+^.

*1-Ethyl-3-[2'-(2'-methoxyphenyl)ethyl]-lH,3H-quinazoline-2,4-dione* (**1bc**). White solid. Yield >95%; mp.128-130°C; IR (NaCl): 2915, 2848, 1701, 1657, 1605, 1483, 1240 cm^-1^; ^1^H-NMR: δ 8.24 (dd, 1H, *J_1_* =1.7, *J_2_* =7.8 Hz, Ar-H), 7.66 (ddd, 1H, *J_1_* = 1.7, *J_2_* =7.3, *J_3_* =8.4 Hz, Ar-H), 7.28-7.16 (m, 5H, Ar-H), 6.85 ( ddd, 1H, *J_1_* =1.0, J_2_ = *J_3_* =8.2 Hz, Ar-H), 4.34 (ddd, 2H, *J_1_* = 5.6, *J_2_* = *J_3_* =7.8 Hz, N-CH_2_), 4.16 (q, 2H, *J* =7.1 Hz, -CH_2_), 3.79 (s, 3H, O-CH_3_), 3.03 (ddd, 2H, *J_1_* =5.8, *J_2_* = *J_3_* =7.4 Hz, Ar-CH_2_), 1.30 (t, 3H, *J* =7.2 Hz, -CH_3_) ppm; ^13^C-NMR: δ 161.6, 150.4, 157.5, 139.1, 134.5, 130.2, 128.8, 127.4, 122.2, 120.1, 112.9, 109.9, 55.1, 41.5, 38.5, 29.6, 12.5 ppm; ESI-MS (m/e): 324.1[M+ H]^+^.

*1-Ethyl-3-[2'-(3'-methoxyphenyl)ethyl]-lH,3H-quinazoline-2,4-dione* (**1bd**). White solid. Yield >94%; mp.112-114°C; IR (NaCl): 2966, 2841, 1701, 1653, 1605, 1483, 1258 cm^-1^; ^1^H-NMR: δ 8.25 (dd, 1H, *J_1_* =1.6, *J_2_* =8.0 Hz, Ar-H), 7.68 (ddd, 1H, *J_1_* = 1.7, *J_2_* =7.3, *J_3_* =8.5 Hz, Ar-H), 7.29-7.18 (m, 4H, Ar-H), 6.91(m, 1H, Ar-H), 6.77 (ddd, 1H, *J_1_* =0.9, *J_2_* = 2.6, *J_3_* =8.2 Hz, Ar-H), 4.31 (ddd, 2H, *J_1_* = 5.6, J_2_ = *J_3_* =7.8 Hz, N-CH_2_), 4.20 (q, 2H, *J* =7.1 Hz, -CH_2_), 3.79 (s, 3H, O-CH_3_), 2.97 (ddd, 2H, *J_1_* =5.6, *J_2_* = *J_3_* =8.4 Hz, Ar-CH_2_), 1.35 (t, 3H, *J* =7.2 Hz, -CH_3_) ppm; ^13^C-NMR: δ 161.6, 150.7, 140.2, 135.0, 131.5, 129.4, 129.1, 122.7, 121.4, 114.2, 113.3, 112.3, 55.1, 43.1, 38.7, 34.0, 12.5 ppm; ESI-MS (m/e): 324.1[M+ H]^+^.

*1-Ethyl-3-[2'-(4'-methoxyphenyl)ethyl]-lH,3H-quinazoline-2,4-dione* (**1be**). White solid. Yield >97%; mp.110-112°C; IR (NaCl): 2974, 2833, 1701, 1657, 1609, 1509, 1483, 1244 cm^-1^; ^1^H-NMR: δ 8.24 (dd, 1H, *J_1_* =1.7, *J_2_* =7.8 Hz, Ar-H), 7.66 (ddd, 1H, *J_1_* = 1.4, *J_2_* = *J_3_* =8.4 Hz, Ar-H), 7.28-7.18 (m, 4H, Ar-H), 6.84(ddd, 2H, *J_1_* =2.2, *J_2_* = *J_3_* =6.6 Hz, Ar-H), 4.31-4.12 (m, 4H, 2 N-CH_2_), 3.77 (s, 3H, O-CH_3_), 2.93 (ddd, 2H, *J_1_* =5.6, *J_2_* = *J_3_* =8.2 Hz, Ar-CH_2_), 1.34 (t, 3H, *J* =7.1 Hz, -CH_3_) ppm; ^13^C-NMR: δ 161.6, 158., 150.3, 139.5, 135.0, 130.7, 129.9, 129.1, 122.7, 115.7, 113.9, 113.3, 55.2, 43.3, 38.7, 33.0, 12.5 ppm; ESI-MS (m/e): 324.1[M+ H]^+^.

*1-Ethyl-3-(3-(4-phenylpiperazin-1-yl)propyl)-*1*H,*3*H-quinazoline-2,4-dione* (**1bf**). White solid. Yield >92%; mp. 94-96°C; IR (KBr): 3010, 2937, 2880, 2803, 1695, 1647, 1604, 1223, 1110 cm^-1^. ^1^H-NMR: δ 8.10 (dd, 1H, *J_1_* =1.6, *J_2_* =7.8 Hz, Ar-H), 7.73 (ddd, 1H, *J_1_* =1.8, *J_2_* =7.4, *J_3_* =8.8 Hz, Ar-H), 7.45 (d, 1H, *J* =8.4 Hz, Ar-H), 7.31-7.14 (m, 3H, Ar-H), 6.89-6.72 (m, 3H, Ar-H), 4.22-4.04 (m, 4H, 2 N-CH_2_), 3.77 (s, 1H), 3.19-2.99 (m, 4H, N-CH_2_), 2.56-2.40 (m, 6H, N-CH_2_), 1.86 (dd, 3H, *J_1_* =7.0, *J_2_* =14.2 Hz, CH_2_), 1.25 (t, 3H, *J* =7.1 Hz, CH_3_) ppm; ^13^C-NMR: δ 164.9, 154.7, 153.7, 143.1, 138.8, 132.5, 131.8, 126.2, 122.6, 119.1, 118.9, 117.7, 59.4, 56.5, 52.1, 51.4, 43.32, 28.0, 16.0 ppm; ESI-MS (m/e): 392.2[M+ H]^+^.
